# A Teething Problem: Artefactual X-Ray Appearances of Odontoid Fracture due to Superimposed Incisor

**DOI:** 10.1155/2012/462865

**Published:** 2012-02-06

**Authors:** Matthew Crocker, Maeve E. Crocker

**Affiliations:** Academic Neurosurgery Unit, St George's University of London, London SW17 0RE, UK

## Abstract

We report a case of artefactual C2 fracture caused by a superimposed incisor tooth in a seven-year-old boy. CT refuted the diagnosis. Trauma clinicians should be aware of this entity to guide correct interpretation of trauma X-rays.

## 1. Introduction

The initial assessment of trauma patients, especially those who are clinically stable or who have been isolated and non-life-threatening injuries, remains based on clinical evaluation supplemented by plain X-rays. In recent years there has been an increase in use of CT earlier in the evaluation of trauma patients due to published guidelines [1]. We report a case of artefactual C2 fracture due to a superimposed incisor tooth resulting in unnecessary transfer of a child and subsequent CT scanning.

## 2. Case Report

**Figure 1:**
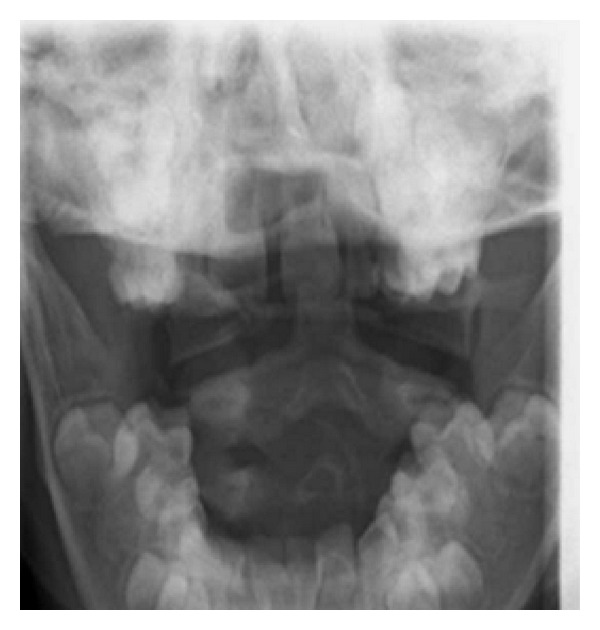
Open-mouth X-ray of the odontoid peg suggesting transverse fracture with lateral displacement through the odontoid peg.

**Figure 2:**
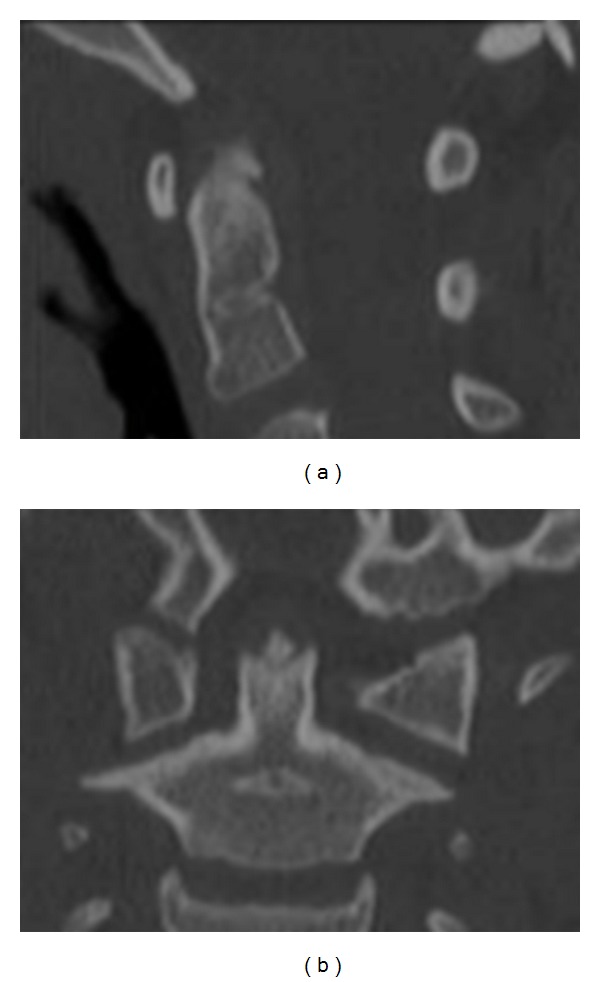
(a) Sagittal and (b) coronal reformatted CT scan confirming normal appearance of the odontoid peg.

A seven-year-old boy was admitted to his local A+E department with head, neck, and chest pain after falling from a tree. He landed on his head and did not lose consciousness. He had walked immediately from the scene and complained of no neurological symptoms. The ambulance service kept him in full cervical spine immobilization. He was fully cooperative with clinical examination, which was normal aside from some mild neck tenderness. X-rays of the cervical spine were performed and the open-mouth odontoid peg view reported as showing a laterally displaced type 2 odontoid fracture (Figure 1). On the basis of this X-ray, he was transferred to the regional neurosciences unit. He remained alert and comfortable. A CT scan with multiplanar reformats of the occiput to C2 was performed showing no evidence of fracture (Figure 2). Review of the open-mouth X-ray showed that the fractured odontoid was a superimposed incisor tooth. He was mobilized and discharged uneventfully. 

## 3. Discussion

This is an illustrative case of a rare but previously described radiographic artifact. In this case there was no iatrogenic harm as a result of the CT imaging, but the patient did undergo an unnecessary interhospital transfer and CT scan due to failure to interpret the X-ray correctly. Interhospital transfer of this patient was undertaken in the belief that the patient would be better served by having his advanced imaging performed in the same department as his definitive treatment. However, in this case it resulted in unnecessary transfer of the patient. This has previously been recognized as a high-risk point of care and therefore remains something to be avoided [2]. The move away from plain X-rays in recent years for some aspects of trauma is the result of various factors. CT of the spine has a very high sensitivity for fractures (approaching 100%) whilst plain X-rays have been found to miss up to 1 in 7 fractures in high-risk patients [1, 3]. With the institution in the UK of trauma networks, there is a heightened awareness of the need to make diagnoses earlier in the management of trauma patients: in addition to other pressures on speed of patient care, there is a stronger mandate for earlier imaging to aid discharge of patients from the emergency department. The result is a loss of familiarity with plain X-ray imaging and clinical assessment and a greater reliance on complex imaging. Therefore in a situation where plain X-rays, together with clinical examination, might be sufficient to exclude serious cervical spine injury, there is a greater chance of the X-rays being interpreted equivocally or incorrectly, resulting in unnecessary CT scanning. This is due purely to lack of familiarity with the imaging. CT remains an imaging modality to be used with caution in children due to the concerns over ionising radiation dose delivered [4]. The ionizing radiation dose of this CT scan as dose-length product (DLP) was 76.77 mGy-cm. This is around one-tenth the dose of a noncontrast CT brain in an adult patient. Whilst this is a low dose, it should still be avoided where possible [5]. There are various strategies being applied in modern trauma centres to reduce the radiation dose associated with CT scanning in children including CT dose modulation which has been shown to provide dose reduction in neurosurgical patients with possible shunt malfunction [6]. Cautionary discussion of educational strategies to ensure repeated, thorough evaluation of plain X-rays or of clinical assessment to prevent unnecessary investigations are going unheard [7].

## 4. Conclusions

An illustrative case of artefactual C2 fracture on X-ray is presented. Clinicians should be familiar with all aspects of plain X-rays in trauma patients as they remain a highly valuable diagnostic investigation.
